# Conservative Treatment of Exposed or Diseased Pulps

**Published:** 1873-06

**Authors:** 


### CONSERVATIVE TREATMENT OF EXPOSED OR DISEASED PULPS.

This was the 6th subject on the programme for discussion before the Mississippi Valley Dental Association.

Dr. Hunt opened the discussion. He said there were two kinds of exposed nerves,—accidental which required very careful treatment, and those exposed for some time, which were of difficult and doubtful treatment.

The first, he would cap and fill at once. The second, he should endeavor to allay the irritation and then cap for a filling, to see if the irritation was allayed. His experience was that a remnant of a nerve is hard to save, but in the main he believes in trying to save. Sometimes it was successful. For capping he used stiptic colloid and oxyde chloride of zinc. To allay irritation he used carbolic acid. If bleeding took place he used full strength and dried out the excess but did not object to a little remaining in. It produced a scab, so to speak, and was soothing to the nerve.

Dr. Rawls took issue with Dr. Hunt on the carbolic acid treatment. He held that it had no properties for allaying inflammation. It simply charred he thought and had no restorative property whatever. When he found the pulps gone so far as to block up the corpuscles, he depleted.

Exposures by accident required no treatment but simply protection.

If inflammation was extensive he advised something milder than carbolic acid, carbolate of glycerine for instance, so as to give nature a chance to recuperate.

Dr. Hunt called Prof. Eames to give his experience with a new remedy for diseased pulps. Prof. Eames responded by a statement about the use of pepsin. He said some claimed success with it by cutting off the diseased part—digesting it away in fact by the use of pepsin. The pepsin was dissolved in water and applied, thus leaving the nerve clean and ready for capping. He had some experience with the new agent and commended its use.

Dr. Frank A. Hunter observed that some of the remarks about oxyde-chloride zinc were plausible but as far as his experience went he found that it would kill nerves. Dr. Knapp stated that he saved ninety-four per cent of his cases. He preferred creasote on the whole, to all other remedies. He saved irritable pulps by waiting till they hurt right bad and then lifting them out.

He found he could not apply oxy-chloride of zinc without hurting. Some pulps would die no matter what the treatment. He closed up with cotton and varnish and sometimes with white wax. It did not make much difference. Exposed pulps could be saved, generally speaking, but if some thought otherwise they had better look to themselves and see if the fault was not with them. He believed the periosteum nourishes the bone and fills up with a new formation. This is often seen under the protection of a little decay that has not been removed and why not under the protection we put there ?

Dr. James Taylor observed that after a practice of thirty odd years, the subject troubled him and he expected it always would.

He knew, to be sure, that there are nerves saved just as a scratch on the hand heals by the first intent, because inflammation does not intervene.

A patient of eighteen years of age has twice the quantity of pulp of one of sixty years of age. As a general thing he did not think applications could be relied upon. What takes place? The dead matter sloughs off and it must be got rid of. He did not want any dead matter between the pulp and the plug; he preferred to lay the gold direct upon the pulp. There was no covering so good as nature's.

He used glycerine to arrest decomposition of the lamina of softened dentine, until nature recuperated.

Nerves are frequently exposed by grinding down of the teeth. The nerves are irritated and nature rushes in to cover them up.

He did not wait until the diseased bone is hardened before filling, and he was satisfied that it was not good practice to cut down to the exposed nerve, but treat the softened dentine and use that as capping.

He had no faith in Oxy-Chloride Zinc, once the nerve is laid bare. Dr. Atkinson claims to make new bone and all that, but Dr. Taylor thought that in two or three years he would find the nerves dead.

He thought failures should be related as much or all the same as successes, for he had seen them following the best operators in the country. He did not believe in discouraging success however, for one in five even to be saved, is an object.

Dr. Rehwinkle considered it a duty to save every nerve possible to be saved. The question with him, however, was to separate the favorable from the unfavorable cases.

If the nerve is inflamed he would not use Escharotics there more than on a boil. To put Chloride-zinc on an exposed nerve would kill it nineteen times out of twenty, but it kills it slowly by drying it up, and because it does this without pain we think it is not done. Yet, on the other hand it has properties which commend it to our use. It is good to mummify the nerve and fill up the vacuum. For extirpating nerves he had faith in pepsin. It was sometimes competent to use arsenic to devitalized pulps. He used very little and left it in twenty-four hours. At the second or third visit he widened the cavity to allow the escape of gas, and, if possible, entangle with the nerve and drew it out, which, if successfully performed, afforded the satisfaction of knowing that there was no dead tissue left. He then kept up an application of iodine to the process until the cavity was left perfectly clean, and the tooth would bear a tap with the hammer.

He made a point of getting rid of all dead tissue, but where the roots are tortuous and difficult he would use Chloride of Zinc.

Dr. Frank A. Hunter desired to know if success depended upon locality or change of climate.

Dr. S. B. Brown said his part of the country was malarious, yet he was successful in saving nerves. In three years observation he found that where pulps were inflamed the use of a poultice of oxide of zinc and creosote were efficacious. He was confident that his successes were ninety-nine per cent.

Dr. Berry observed that Dr. Watt had affirmed that Oxychloride of Zinc would kill every nerve.

Dr. Clancy here read a brief paper.

Dr. Bradley thought application of oxy-chloride of zinc to the pulps wrong. He first stopped pain with acetate of lead, then made a fine paste of oil of cloves, peppermint, rosemary, creosote and morphine, with which he covered the nerve carefully and pressed out the excess with a piece of spunk. If the nerve is very tender he put on a thick coat, and then applied the oxy-chloride of zinc. If there was very much pain he usually sent the patient away. He only lost three nerves in seventy-five since last fall. In cases of sore teeth he used the paste and carbolic acid, then filled with Hill's Stopping.

Discussion closed.

